# Supplementary figures and images for: Oropouche fever outbreak in Brazil: Key factors behind the largest epidemic in history
Source: PLoS One. 2025 Jul 28;20(7):e0327845. doi: 10.1371/journal.pone.0327845 (PMC12303302; doi:10.1371/journal.pone.0327845)

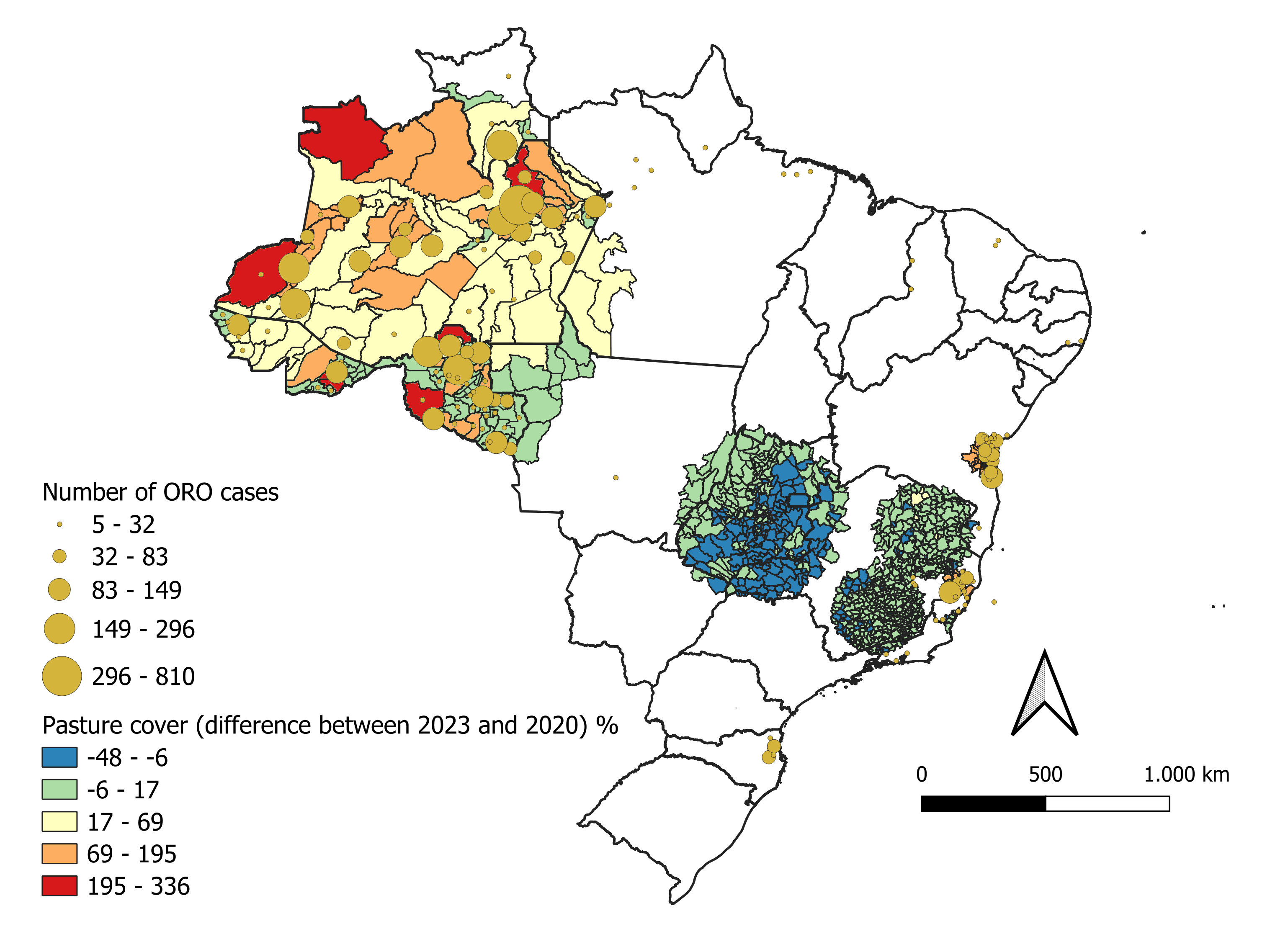

Supplement: S1 Fig — The map was built using ArcGIS version 9.1. Source of map base layers: IBGE: https://www.ibge.gov.br/geociencias/cartas-e-mapas/mapas-estaduais.html. Open-source CC BY 4.0 license. (PNG) [file pone.0327845.s002.png]
